# The pharmacogenomic landscape of an Indigenous Australian population

**DOI:** 10.3389/fphar.2023.1180640

**Published:** 2023-05-22

**Authors:** Sumudu Rangika Samarasinghe, Wendy Hoy, Sudhir Jadhao, Brendan J. McMorran, Henk-Jan Guchelaar, Shivashankar H. Nagaraj

**Affiliations:** ^1^ Centre for Genomics and Personalised Health, Queensland University of Technology, Brisbane, QLD, Australia; ^2^ Faculty of Medicine, University of Queensland, Brisbane, QLD, Australia; ^3^ John Curtin School of Medical Research, College of Health and Medicine, Australian National University, Canberra, ACT, Australia; ^4^ Department of Clinical Pharmacy and Toxicology, Leiden University Medical Center, Leiden, Netherlands; ^5^ Translational Research Institute, Queensland University of Technology, Brisbane, QLD, Australia

**Keywords:** pharmacogenomics, pharmacogenetics, adverse drug reactions, population genomics, Indigenous Australians, variable drug response

## Abstract

**Background:** Population genomic studies of individuals of Indigenous ancestry have been extremely limited comprising <0.5% of participants in international genetic databases and genome-wide association studies, contributing to a “genomic gap” that limits their access to personalised medicine. While Indigenous Australians face a high burden of chronic disease and associated medication exposure, corresponding genomic and drug safety datasets are sorely lacking.

**Methods:** To address this, we conducted a pharmacogenomic study of almost 500 individuals from a founder Indigenous Tiwi population. Whole genome sequencing was performed using short-read Illumina Novaseq6000 technology. We characterised the pharmacogenomics (PGx) landscape of this population by analysing sequencing results and associated pharmacological treatment data.

**Results:** We observed that every individual in the cohort carry at least one actionable genotype and 77% of them carry at least three clinically actionable genotypes across 19 pharmacogenes. Overall, 41% of the Tiwi cohort were predicted to exhibit impaired CYP2D6 metabolism, with this frequency being much higher than that for other global populations. Over half of the population predicted an impaired CYP2C9, CYP2C19, and CYP2B6 metabolism with implications for the processing of commonly used analgesics, statins, anticoagulants, antiretrovirals, antidepressants, and antipsychotics. Moreover, we identified 31 potentially actionable novel variants within Very Important Pharmacogenes (VIPs), five of which were common among the Tiwi. We further detected important clinical implications for the drugs involved with cancer pharmacogenomics such as thiopurines and tamoxifen, immunosuppressants like tacrolimus and certain antivirals used in the hepatitis C treatment due to potential differences in their metabolic processing.

**Conclusion:** The pharmacogenomic profiles generated in our study demonstrate the utility of pre-emptive PGx testing and have the potential to help guide the development and application of precision therapeutic strategies tailored to Tiwi Indigenous patients. Our research provides valuable insights on pre-emptive PGx testing and the feasibility of its use in ancestrally diverse populations, emphasizing the need for increased diversity and inclusivity in PGx investigations.

## Introduction

Global estimates suggest that less than 0.5% of all participants within international genetic databases and genome-wide association studies (GWAS) are of Indigenous ancestry. As of 2018, 22% of genetic data in GWAS were derived from persons of non-European ancestry, while less than 4% were contributed by Africans, Latinos, and Indigenous individuals (Sirugo et al., 2019). In 2019, the overall Indigenous participation in GWAS cohorts was just ∼0.02%. A recent study by Hitchman et al. have reported a rare uncertain function allele CYP2D6*71, with a high prevalence of 8.9% among New Zealand Māori and Pacific peoples demonstrating the need for more genetic studies in distinct ethnic groups (Hitchman et al., 2022). The lack of reference variant data for these minority groups has hampered clinical variant analysis efforts focused on these populations, resulting in the imprecise interpretation of clinical results and thereby directly impacting the access of these populations to equitable health outcomes (Garrison et al., 2019).

Indigenous Australians represent the world’s longest continuously surviving cultural group, having inhabited the Australian continent for over 60,000 years. Their culturally and ancestrally diverse communities have descended from a single founding population roughly 10,000–32,000 years ago. There are about 600 distinct Indigenous Australian groups, each with their own culture, customs, language, and laws (Malaspinas et al., 2016). However, these Indigenous groups face disproportionately high burdens of disease and mortality compared to other Australians across all age groups. Non-communicable diseases such as chronic lung disease, cardiovascular disease, type-2 diabetes, chronic renal failure, cancer, and mental disorders are the major contributors to the high rates of mortality among adult members of the Indigenous population. The burden of chronic diseases in this population imposes extensive suffering, social and familial dislocation, and burdensome healthcare costs on these Indigenous patients and their communities (Thynne and Gabb, 2016). Tiwi people are an Indigenous group living on Bathurst and Melville Islands, close to Darwin in Australia. There are 2348 individuals live in Tiwi Islands in comparative isolation distinct from mainland Aboriginal people (Australian Bureau of Statistics, 2021) and represents a highly homogeneous population. In this study, we focus on the Pharmacogenomics (PGx) in this distinct Indigenous group.

Adverse drug reactions (ADRs), inadequate efficacy, and treatment failure are common, yet serious, consequences of trial-and-error prescription efforts globally. The likelihood of an unsuitable prescription may be >80% in some instances, with an up to 40% risk of an ADR (Cacabelos et al., 2019). ADRs have been attributed to increased morbidity, mortality, and additional costs that can affect overall patient wellbeing in addition to placing a significant burden on healthcare systems and the pharmaceutical industry. A prevalence of 6.5% has been reported for ADR-related hospital admissions, with a median bed-stay of up to 8 days and an estimated annual cost of USD 847 million for the National Health System in England (Pirmohamed et al., 2004). ADRs have also been reported as the fourth leading cause of death in the United States and Canada (Hacker, 2009). Furthermore, 462 drugs have been withdrawn from the market between 1953-2013 due to associated severe ADRs (Onakpoya et al., 2016). In Australia, nearly 1.7 million patients were prescribed a medication listed by the Clinical Pharmacogenetics Implementation Consortium (CPIC, 2022) in 2017, 40% of which were predicted to carry one or more PGx variants associated with the prescribed medication (Polasek et al., 2019). Moreover, 250,000 annual hospital admissions in Australia were medication related with an estimated cost of AUD 1.4 billion to the healthcare system, of which two-thirds were potentially preventable (Lim et al., 2022). The genetic profile of an individual is estimated to account for 30%–40% of their response to medications (Kozyra et al., 2017). Much like this inter-individual variability, significant differences in the distribution of variations and associated drug response outcomes across racially and ethnically diverse groups are well established (Zhou and Lauschke, 2021).

Genetic and clinical evidence to support inter-individual and inter-ethnic diversity in drug responses, and PGx has evolved as an effective tool to manage medication risk. Evidence-based guidelines for a total of 147 drugs are published by the CPIC, the Dutch Pharmacogenetics Working Group (DPWG) (KNMP Pharmacist organization Guidelines, 2022), and other professional societies. A total of 411 FDA approved drug label annotations are available for 367 unique drugs to assist in clinical decision-making (U. S. Food and Drug Administration, 2022). Myriad PGx information is easily accessible from resources including PharmGKB (PharmGKB, 2022) and PharmVar (PharmVar, 2022). However, PGx research and clinical trials are biased in their representation, focusing largely on populations of European ancestry and exhibiting inappropriate inclusion of other racial/ethnic groups. Therefore, generalizing the PGx findings of these studies in genetically distinct minority groups can be problematic (Thynne and Gabb, 2016). The Caucasian ethnicity is the most prevalent among the general Australian population and vary from Indigenous Australians. The AFs of *CYP2D6*, *CYP2C9*, *CYP2C19*, and *VKORC1* in a cohort of Australian patients were consistent with the previously reported frequencies in Caucasians (Mostafa et al., 2019). Despite numerous attempts to characterize the unique PGx profiles of genetically distinct populations (Zhou and Lauschke, 2018; Gonzalez-Covarrubias et al., 2019; Sahana et al., 2022) their representation in PGx research remains limited. Indigenous Australians harbor genetic diversity seen in few other population groups. Although the drug safety information among these people are highly limited, previous evidence show that they carry a high ADR risk for certain medications. In one clinical case report, a 30-year-old Australian indigenous woman carrying a CYP2C9*3 poor metabolizer genotype and an HLA-B*56-02 positive genotype experienced a phenytoin-induced drug rash with eosinophilia and other systemic symptoms (Somogyi et al., 2019). Also, serious statin-associated toxicities have been reported among Indigenous Australians (Gabb et al., 2013), indicating the greater importance of pre-emptive testing for potential ADR risk in this patient population. However, Tiwi Indigenous people, like many other ethnicities within Australia and globally, are severely underrepresented in population genomic studies conducted to date, resulting in a significant “genomic gap” that limits their access to personalised medicine and equitable healthcare.

We previously conducted one of the earliest Indigenous Australian PGx studies evaluating pharmacological variability in this population (Jaya Shankar et al., 2022). Building on this recent report, we herein carried out a comprehensive PGx analysis of a founder Indigenous population in Australia, harboring a unique genetic architecture. Specifically, we analysed whole genome sequencing (WGS) data and associated pharmacological treatment data from 473 Tiwi individuals, including the previously analyzed 187 participants to identify key variants of pharmacological relevance enriched within the population that could potentially interfere with medication use in the context of chronic disease. This study provides a better understanding of the underlying genetic factors in the Tiwi population and highlights the critical need for robust strategies aimed at implementing pharmacovigilance to improve the health outcomes for members of this Indigenous community.

## Materials and methods

### Study population and datasets

The WGS data for this study were acquired from blood samples collected from 492 Tiwi individuals between 2013 and 2014 in a previous study conducted to investigate the prevalence of chronic kidney disease (CKD) in this population (Thomson et al., 2019). The associated meta-data included: lifestyle information, CKD status, comorbidities, medication, blood glucose, blood pressure, blood albumin, and creatinine. The cohort was sequenced in four batches using PCR-free sample preparation and Illumina Novaseq6000 paired-end sequencing with an average coverage 30x. After quality control (QC) and filtering, 459 samples were retained for further downstream analysis. The GRCh38 version of multi-sample VCFs from the 1000 Genome phase three project (1KGP) (*n* = 2504) (1000 Genomes Project Consortium et al., 2015) was used in the PCA analysis.

### Indigenous community consultations

This project has been carried out in consultation and ongoing engagement with community experts, Indigenous Elders and lead Indigenous research experts. Guidelines developed to guide the ethical conduct of research with Indigenous peoples have been adhered to in all aspects (e.g., NHMRC). The core values of Spirit and Integrity, Cultural Continuity, Equity, Reciprocity, Respect and Responsibility have been embedded throughout the project ***.*** Participants provided consent for their DNA samples to be used to investigate the causes of CKD (Thomson et al., 2019). The current study subsequently received the support of the Tiwi Island Land Council.

### Ethical approval

Approval was formally obtained through several consultations over 30 years with the Tiwi Land Council, Tiwi Elders, and traditional owners of the Tiwi Islands. The Tiwi Islands are privately owned and follow a permission system that recognises the importance and value of Aboriginal responsibility towards the country and is consistent with land titles held under Australian Law. The Northern Territory Department of Health also provided ethical approval for this study (2012–1767) followed by approvals by the QUT HREC (No. 2022-6199-10439- HE26) and ANU HREC (No. 2014-663). Ethics for human participants were reviewed by the human research ethics committees of The Northern Territory Department of Health (2012–1767), The Australian National University (2014-663), The University of Queensland (2012001146), and The University of Tasmania (H0012832).

### Sequence and variant pre-processing and quality control

Sequenced reads from 473 samples were mapped to GRCh38 reference genome and variant calling was carried out according to GATK best practices using the DRAGEN-GATK pipeline v3.8.4 (Miller et al., 2015). Multi-sample VCF files for 473 Tiwi individuals were pre-processed using BCFtools v1.9 (Danecek et al., 2021) to normalize and left-align the indels, add unique variant IDs, and remove exact duplicate variants. Genotype-level filtering was performed using BCFtools (DP < 10 and/or GQ < 20 genotypes were set to missing). Filtered per-chromosome VCFs were converted to the plink format using plink v1.9 (Chang et al., 2015). Variants with >10% missingness were excluded and Principal Component Analysis (PCA) was performed with stringently filtered high-quality autosomal single nucleotide polymorphisms (SNPs) to identify outlier samples. Duplicate samples and monozygotic twins were removed using plink–king-cutoff 0.354. Samples with extreme heterozygosity (±3 standard deviations from the mean heterozygosity rate) and missingness >10% were also excluded. Variants with Hardy-Weinberg Equilibrium (HWE) *p*-value < 1 × 10^−6^ were removed to obtain a high-quality variant set for downstream analyses.

### Principal Component Analysis

Autosomal SNPs were selected from the VCF file. Variants were filtered for >1% missingness, MAF <1%, and HWE P < 1 × 10^−6^. Samples with >10% missingness were excluded. Filtered variants were LD-pruned using plink–indep-pairwise 50 5 0.2 and duplicate/monozygotic twins were removed using plink–king-cutoff 0.354. The 1KGP multi-sample VCF file containing 2504 unrelated individuals (Americans: 347, Europeans: 503, Africans: 661, South Asians: 489, East Asians: 504) was also filtered using the above criteria, and common variants in both the Tiwi and 1KGP datasets were merged. PCA was run using plink–pca and the eigenvec file was plotted using R statistical software v.4.0.3 (R: A language and environment for statistical computing, 2021). PCA outlier samples were identified using PC1 and PC2 thresholds in PCA plots.

### Identification of star alleles

Haplotypes consisting of a combination of variants [SNPs, indels, or structural variants (SVs)] that are inherited together are called star alleles (*) in pharmacogenomics. We selected 19 genes with pharmacological relevance for this analysis and a set of bioinformatics tools to identify star alleles present in these genes. The star allele calling tools included Cyrius v1.1.1 (Chen et al., 2021), PyPGx v0.17.0, StellarPGx v1.2.5 (Twesigomwe et al., 2021), Stargazer v.1.0.8 (Seung-been Lee et al., 2019; Lee et al., 2019), Aldy v3.3 (Numanagic et al., 2018) and PGxPOP v1.0 (McInnes et al., 2021). A consensus set of star alleles, called in agreement with at least two tools per each gene, were identified through an ensemble genotyping approach for further downstream analysis (Twesigomwe et al., 2020), except *ABCG2*, *RYR1* and *CACNA1S* which are only supported by PyPGx tool (Lee et al., 2022). Genes with known SVs such as large deletions, insertions, or hybrids (e.g., *CYP2D6*, *CYP2B6*, *CYP4F2*, and *G6PD*), were evaluated using Cyrius, PyPGx, and StellarPGx which have better capability to detect complex variants. The GRCh38-aligned BAM files were used as input to the tools and consensus genotypes were manually evaluated.

Metabolic phenotypes [normal metabolizer (NM), intermediate metabolizer (IM), poor metabolizer (PM), rapid metabolizer (RM) or ultra-rapid metabolizer (UM)] and phenotypes associated with altered function in drug targets or drug transporters were assigned for diplotypes according to PharmGKB, PharmVar and CPIC definitions. Population specific frequencies for star alleles and phenotypes were assigned by PharmGKB.

### Prevalence of high-evidence variants

PharmGKB clinical annotations for 265 unique variants (118 SNPs and 147 haplotypes) with PharmGKB level 1A and 1B were considered as high-evidence PGx variants (accessed 10th July 2022), and were screened for their frequency and distribution in the Tiwi and compared to other global populations from gnomAD, 1KGP and UK Biobank (UKB) 200k exome data (Szustakowski et al., 2021).

### Characterization of potentially actionable variants in VIPs

**FIGURE 1:**
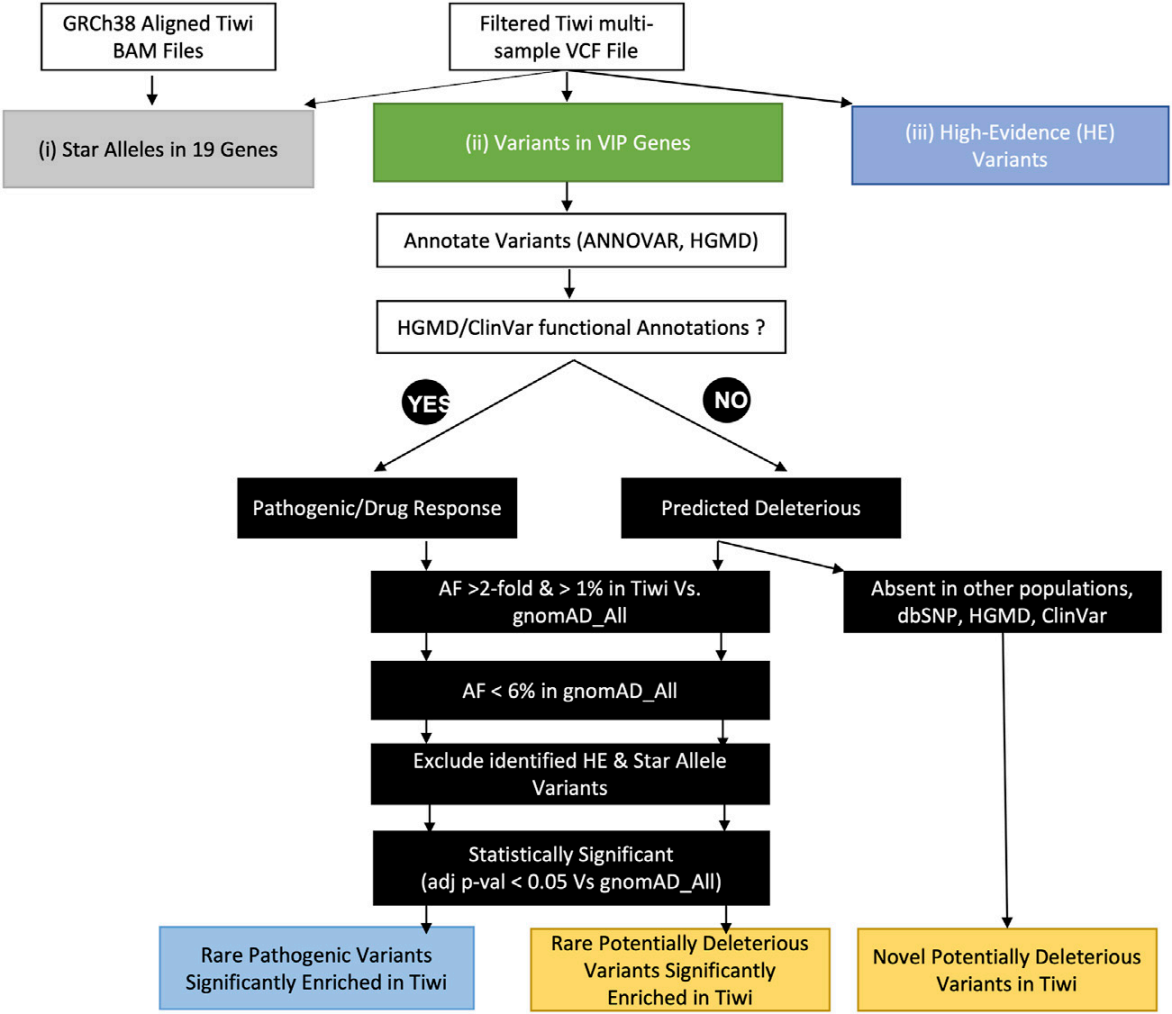
Schematic diagram of downstream analyses to identify pharmacologically important variants in the study population. This analysis was performed through three approaches: 1) the identification of star alleles in 19 pharmacogenes using an ensemble of star allele calling tools, 2) the identification of pharmacologically important variants in VIP genes, and 3) the identification of high-evidence variants in the study cohort.

We screened 67 VIPs (*MT-RNR1* mitochondrial gene was excluded), defined by the PharmGKB as significantly involved in the metabolism or response of one or more drugs, to identify known and novel deleterious variants enriched in Tiwi that could potentially alter drug responses that have yet to be included in official star allele definitions (Figure 1). The variants in 67 genes were selected from the filtered Tiwi VCF and annotated with ANNOVAR (Wang et al., 2010) to add global population (1KGP, gnomAD) AFs, dbSNP rsIDs, and ClinVar annotations. Additionally, clinical annotations from the Human Gene Mutation Database (HGMD) (Stenson et al., 2003) and AFs from the UKB 200k exome data were also added. AF comparisons between Tiwi and gnomAD_All population were carried out using Fisher’s exact test with Benjamini–Hochberg correction for multiple testing error to select statistically significant variants. Variants with functional annotations in ClinVar or HGMD were screened to identify known pathogenic variants in the Tiwi population (Figure 1). Variants without any functional information were assessed using *in silico* computational algorithms (SIFT “D”, PolyPhen2 “D” or “P”, LRT “D”, MutationTaster “D” or “A”, MutationAssessor “H” or “M”, FATHMM “D”, MetaSVM “D”, MetaLR “D” or CADD >20). Variants predicted to be deleterious by at least two tools or having a CADD phred score >20 were defined as potentially deleterious that could alter the protein function. Of these, commonly enriched variants (AF > 1%) with >2-fold AF in Tiwi Vs. rare in gnomAD_All (AF < 6% in gnomAD_All) or absent in all other global populations were identified. The AF comparisons between Tiwi and gnomAD_All population were carried out using Fisher’s exact test with Benjamini–Hochberg multiple testing error correction. Variants having AF differences with adjusted *p*-value <0.05 vs. gnomAD_All were selected as statically significant variants for further downstream analysis. High-evidence variants and variants detected in the star allele analysis were excluded to identify potentially actionable novel PGx variants in VIPs.

## Results

### Population structure of Tiwi people

**FIGURE 2:**
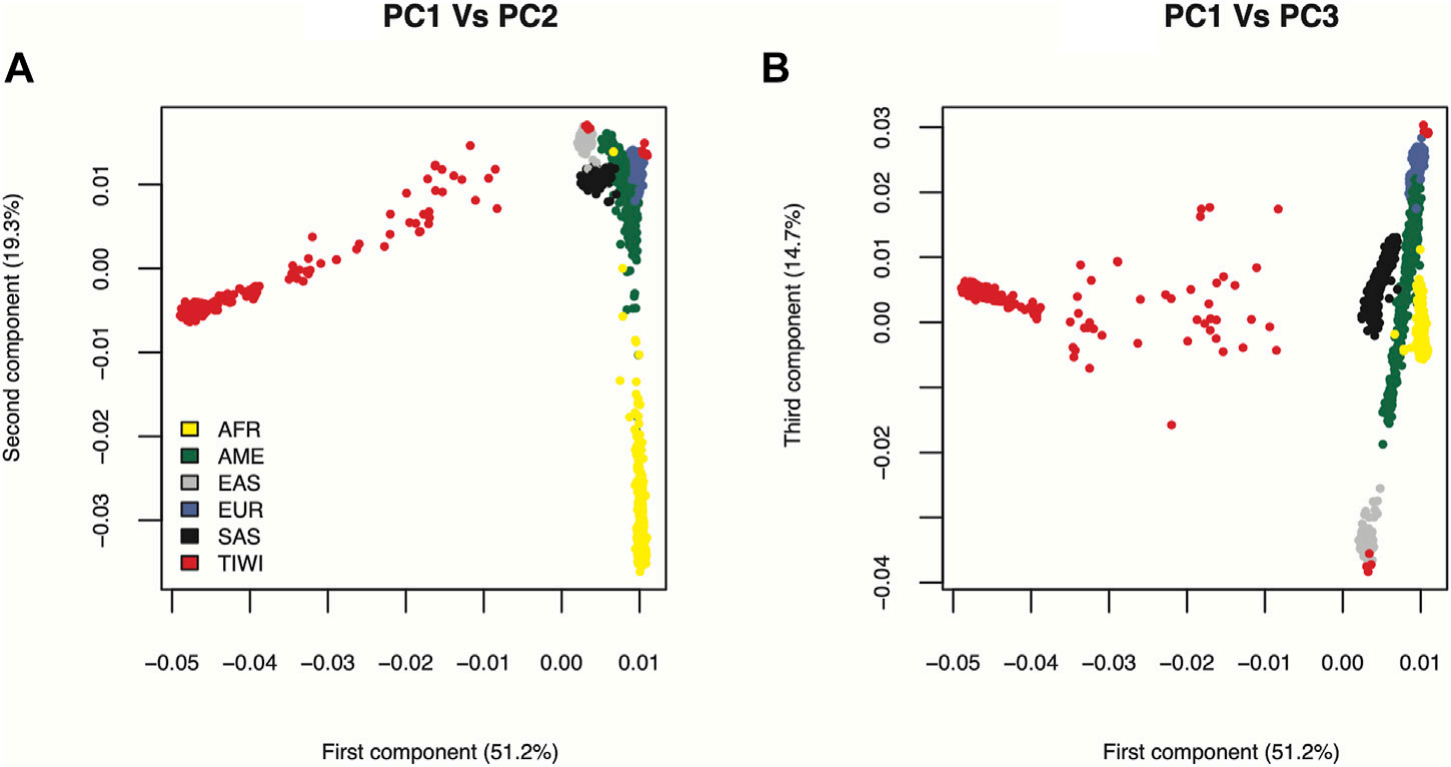
PCA plot of Tiwi and 1KGP global populations. The principal components were generated using stringently filtered 54,710 common genome-wide SNPs from 469 Tiwi and 2504 1KGP unrelated individuals. **(A)** Principal components 1 and 2 explained 70.5% of the observed variance **(B)** Principal components 1 and 3 explained 65.9% of the observed variance. African (AFR), American (AME), East Asian (EAS), European (EUR), South Asian (SAS).

We have previously shown that Tiwi people are genetically distinct using a separately collected cohort of 249 individuals (Thomson et al., 2019) and a subset of the cohort used in this study consisting of 187 individuals (Jaya Shankar et al., 2022). The findings were recapitulated in the current study through PCA analysis of a larger cohort consisting of 469 individuals after QC (Figure 2). A total of ten individuals clustered with the European and East Asian populations likely due to admixture in the Tiwi population. These individuals were excluded from our analyses to remove bias in allele frequency calculations. Majority of the Tiwi people clustered separately from other global populations, revealing that they are genetically distinct.

### Distribution of clinically actionable haplotypes, genotypes and phenotypes in Tiwi people

#### The prevalence of no function alleles and their clinical implications

**FIGURE 3:**
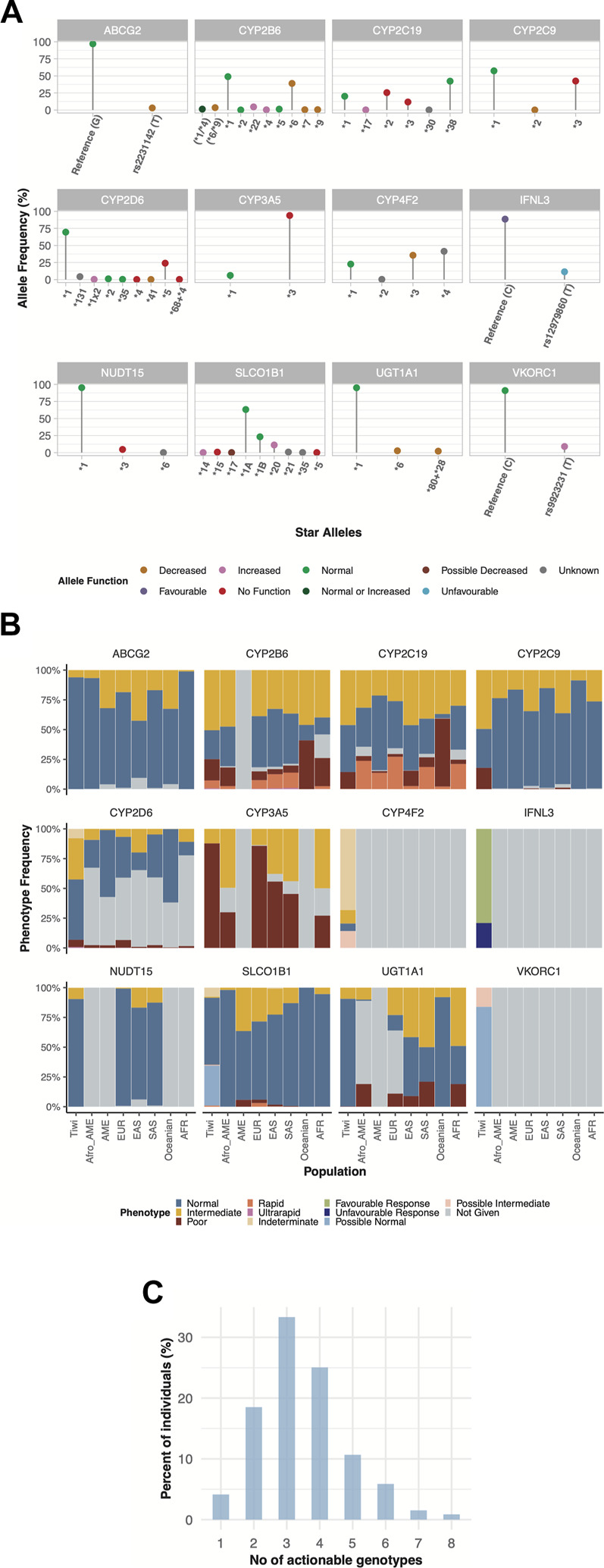
Pharmacogenetic variability of haplotypes (star alleles) and predicted phenotypes across twelve pharmacogenes in Tiwi. **(A)** Allele frequencies of star alleles identified across 12 genes in the study cohort. Colours on dots in star alleles represent the allele function. **(B)** Frequency and distribution of predicted phenotypes associated with star alleles compared to global populations across 12 pharmacogenes. **(C)** Distribution of clinically actionable genotypes in Tiwi. African American (Afro_AME), American (AME), European (EUR), East Asian (EAS), South Asian (SAS), African (AFR). *In* **(A)** : (*6/*9)—could not accurately assigned between *6 and *9. (*1/*4) - could not accurately assigned between *1 and *4.

No function alleles in certain pharmacogenes resulting in an impaired metabolic phenotype were found to be more common in the Tiwi cohort compared to other populations (Figure 3A, Supplementary Table S1). For instance, the CYP3A5*3 was the most prevalent allele with a frequency of 0.94. A similar frequency has been reported in Europeans (0.92) whereas the frequency tends to be lower in others (South Asians: 0.67 and Africans: 0.24). The CYP3A5 IMs/expressers (*1/*3) and PMs/nonexpressers (*3/*3) were 12.2% and 87.8% in the cohort, respectively. These prevalences closely represent frequencies in Europeans (IMs: 14%; PMs: 86%) but the presence of PMs in other populations were comparatively lower (<77%) while IMs were higher than in Tiwi (>21%) (Figure 3B, Supplementary Table S1). Unlike other CYP enzymes, CYP3A5 expressers (NMs or IMs) have been recommended to use a higher starting dose of tacrolimus, while a standard starting dose has been recommended for nonexpressers (PMs) (Birdwell et al., 2015) (Table 1). Tacrolimus concentration should be high enough to prevent organ rejection while not reaching toxic levels. The presence of expressers in the Tiwi was low compared to other populations. However, due to substantial evidence of kidney-related complications which could lead to kidney transplant events, genotyping could be beneficial to increase tacrolimus effectiveness and prevent ADRs in this population.

**TABLE 1:** Clinically actionable genotypes and phenotypes commonly detected in the Tiwi population and their clinical implications.

Gene	Common genotypes in Tiwi (>1%)	Common phenotypes in Tiwi (>1%)				Affected drug/ drug class with clinical guidelines	Condition for the use of drug	Affected genotype and consequences	Clinical guidelines recommended by the CPIC/DPWG
		NM	IM	PM	RM/UM				
*CYP2D6*	*1/*1, *1/*131, *1/*2, *1/*5, *5/*131, *5/*5	50.7	34.6	6.2	<1	TCAs (amitriptyline, nortriptyline, clomipramine, doxepin, imipramine)	Depression, obsessive-compulsive disorder (OCD), neuro-pathic pain, migraine prophylaxis	IMs and PMs: Markedly reduced metabolism and higher risk for cardiotoxicity	Reduced doses, avoid TCAs or use an alternative drug not primarily metabolized by CYP2D6 Hicks et al. (2017). DPWG recommends reduced doses for all TCAs with close monitoring in IMs and PMs^b^
						SSRIs (paroxetine, fluvoxamine)	Major depressive and anxiety disorders	IMs and PMs: Reduced metabolism and higher risk of side effects	Reduced doses, select an alternative drug not primarily metabolized by CYP2D6 Hicks et al. (2015)
						Antidepressant (Venlafaxine)	Depression, social anxiety disorder, and cataplexy	IMs and PMs: Increased risk of side effects and reduced efficacy	DPWG recommends reduced doses, alternate drugs and monitor for side effects in IMs and PMs^b^
						Atypical antipsychotics (aripiprazole, brexpiprazole), Typical antipsychotics (haloperidol, pimozide, risperidone, zuclopenthixol)	Schizophrenia, bipolar disorder, add-on treatment in major depressive disorder	PMs: Increased risk of side effects	DPWG recommends reduced standard maximum doses of aripiprazole, brexpiprazole, risperidone and haloperidol in PMs, reduced standard maximum doses of pimozide in IMs and PMs and reduced zuclopenthixol doses or alternate in IMs and PMs^b^
						Opioids (codeine and tramadol)	Pain management	IMs and PMs: Ineffective analgesia and adverse reactions	Use an alternative drug Crews et al. (2021). DPWG recommends alternative drug, increase doses, and monitor for inadequate analgesia^b^
						Tamoxifen	Antiestrogen used in the breast cancer therapy	IMs and PMs: Reduced activity	Use an alternative hormonal therapy Goetz et al. (2018). DPWG recommends a dose increase or an alternative in IMs and PMs^b^
						Atomoxetine	Nonstimulant used to treat attention-deficit-hyperactivity disorder (ADHD)	IMs and PMs: Reduced activity, risk of side effects	Use reduced doses Brown et al. (2019). DPWG recommends reduced dose if side effects occur^b^
						Metoprolol (ß-blockers)	Hypertension, angina, myocardial infarction, atrial fibrillation/flutter	IMs and PMs: Reduced activity, occurrence of asymptomatic bradycardia	DPWG recommends smaller steps in dose titration and/or prescribe no more than 25% of the standard dose^b^
						Eliglustat	Long-term treatment of type 1 Gaucher disease	IMs and PMs: Reduced activity, risk of side effects	DPWG recommends alternative drug or reduced dose and be alert of side effects^b^
						Antiarrhythmic drugs (flecainide, propafenone)	Used to prevent and treat abnormally fast heart rates	IMs and PMs: Increased risk of side effects	DPWG recommends reduced doses in IMs and PMs^b^
*CYP2C9*	*1/*1, *1/*3, *3/*3	32.7	49.5	17.9	ND	NSAIDs (meloxicam, piroxicam, tenoxicam, celecoxib, flurbiprofen, ibuprofen, lornoxicam)	Commonly used analgesics for chronic/acute pain or musculosketal injuries	IMs and PMs: High risk for toxicities related to NSAIDs, e.g., gastrointestinal bleeding, hypertension, myocardial infarction, heart failure, renal damage, etc.	Initiate therapy with lowest/reduced doses for shorter duration, close monitoring for ADRs or alternate/combination therapy to avoid serious ADRs based on the type of NSAID Theken et al. (2020)
						Phenytoin	Antiepileptic drug used to prevent and treat seizures	IMs and PMs: Reduced phenytoin metabolism and probability of toxicities with phenytoin use	Adjust doses by close monitoring for drug response and side effects Karnes et al. (2021). DPWG recommends reduced standard doses and close monitoring for ADRs^b^
						Statin (Fluvastatin)	Reduce cholesterol and risk for cardiovascular disease	IMs and PMs: Increased myopathy risk upon statin (fluvastatin) use	Reduced doses/adjust doses, consider alternate statin/combination therapy Cooper-DeHoff et al. (2022)
						Warfarin	Thromboembolic disorders	IMs and PMs: Bleeding upon warfarin therapy	Pharmacogenetic dose algorithms to select initial or maintenance warfarin dose Johnson et al. (2017). DPWG recommends reduced doses in IMs and PMs and using dosing algorithms^b^
						Siponimod	To treat relapsing multiple sclerosis	IMs and PMs: Increased risk of side effects	DPWG recommends reduced doses in IMs and avoid use of drug in PMs^b^
*CYP2C19*	*1/*1, *1/*2, *1/*3, *1/*38, *2/*38, *2/*2, *2/*3, *3/*3, *3/*38, *38/*38	39.4	46	14.2	<1	Clopidogrel	Common antiplatelet drug used to reduce the risk of myocardial infarction and stroke	IMs and PMs: Reduced clopidogrel action, high risk of adverse cardiac and cerebrovascular events upon clopidogrel therapy	Use an alternative for clopidogrel such as prasugrel or ticagrelor Lee et al. (2022a). DPWG recommends avoiding clopidogrel and using alternatives in IMs and PMs^b^
						TCAs (imipramine)	Depression, OCD, neuro-pathic pain, migraine prophylaxis	PMs: Markedly reduced metabolism and higher risk of side effects	Reduced doses, avoid TCAs and use an alternative drug not metabolized by CYP2C19 Hicks et al. (2017). DPWG recommends reduced imipramine in PMs and alternatives in UMs for OCD indication^b^
						SSRIs (citalopram, escitalopram, sertraline)	Major depressive and anxiety disorders	IMs and PMs: Reduced metabolism and higher risk of side effects	Reduced doses, select an alternative drug not metabolized by CYP2C19 Hicks et al. (2015). DPWG recommends not to exceed the recommended doses of citalopram, escitalopram in IMs and PMs and sertraline in PMs^b^
						PPIs (omeprazole, lansoprazole, dexlansoprazole)	Gastroesophageal reflux disease, gastric and duodenal ulcers, erosive esophagitis, *Helicobacter pylori* infection, and pathological hypersecretory conditions	IMs and PMs: Potential increased efficacy with increased toxicity	Reduced dose after achieving therapeutic efficacy and close monitoring Lima et al. (2021)
						Voriconazole	Antifungal agent	PMs: High risk of adverse events	Use of an alternative drug not predominantly metabolized by CYP2C19 Moriyama et al. (2017). DPWG recommends reduced standard doses in PMs and close monitoring in IMs and PMs^b^
*VKORC1*	C/T, T/T (Reference C and variant T allele with rs9923231)	Normal function: 83.9, Possible decreased function: 14.2, Decreased function: 2				Anticoagulants (warfarin, acenocoumarol, phenprocoumon)	Thromboembolic disorders	Decreased function phenotypes: Significant association with warfarin sensitivity	Require lower warfarin doses Johnson et al. (2017). DPWG recommends reduced standard initial dose of warfarin, acenocoumarol and phenprocoumon with close monitoring in T/T (-1639 AA genotype)^b^
*CYP4F2* *^a^*	*1/*1, *1/*3, *3/*3, *3/*4, *4/*4, *1/*4	Normal function: 6.3, Possible decreased: 14.2, Decreased function: 11.3				Warfarin	Thromboembolic disorders	Decreased dose phenotypes: Affected warfarin response	Require higher warfarin doses Johnson et al. (2017)
*CYP3A5*	*1/*3, *3/*3	ND	12.2	87.8	ND	Tacrolimus	Immunosuppressant used to prevent organ rejection	IMs: Associated with tacrolimus response	Increased/adjusted starting doses Birdwell et al. (2015). DPWG recommends increased doses in IMs specially in liver transplant patients and donors^b^
*CYP2B6*	*1/*1, *1/*22, *1/*5, *1/*6, *6/*22, *6/*6, *1/*9, *4/*9	24.3	50.5	17.9	7.2	Efavirenz	Widely used in the antiretroviral treatment in HIV type-1 infection	IMs and PMs: Increased risk of central nervous system adverse events	Initiate efavirenz treatment with reduced doses Desta et al. (2019). DPWG recommends adjusting doses of efavirenz in IMs and PMs^b^
*NUDT15*	*1/*1, *1/*3	90.4	9.4	ND	ND	Thiopurines (mercaptopurine, azathioprine, thioguanine)	Used to treat malignant and non-malignant immunological disorders	IMs: High risk of thiopurine related adverse events e.g., leukopenia, neutropenia, myelosuppression	Start treatment with reduced doses of drug Relling et al. (2019). DPWG recommends reduced mercaptopurine, azathioprine and thioguanine doses in IMs and reduced or alternate doses in PMs^b^
*UGT1A1*	*1/*1, *1/*6, *1/*80+*28	90.4	9.4	<1	ND	Atazanavir	Antiretroviral protease inhibitor	PMs:. Markedly decreased activity and high risk of jaundice	Consider an alternative drug Gammal et al. (2016)
*ABCG2*	G/G, G/T (Reference G and variant T allele with rs2231142)	Normal function: 93.9, Decreased function: 6.1				Statins (rosuvastatin)	Reduce cholesterol and cardiovascular disease	Decreased function phenotypes: Increased drug exposure compared to normal, myopathy risk unknown, increased lipid-lowering effects	Desired starting dose, adjust doses based on disease and ancestry guidelines Cooper-DeHoff et al. (2022)
						Allopurinol	Decrease high blood uric acid levels	Decreased function phenotypes: Reduced excretion of uric acid	DPWG recommends increased allopurinol doses in G/T genotypes^b^
*SLCO1B1*	*1A/*1A, *1A/*1B, *1A/*20, *1B/*1B, *1B/*20	Normal function: 56.1, Possible normal function: 33.6, Decreased function: 1, Possible decreased function: 1, Poor function: <1, Increased function: 1				Statins (atorvastatin, fluvastatin, lovastatin, pitavastatin, pravastatin, rosuvastatin, simvastatin)	Reduce cholesterol and cardiovascular disease	Decreased and Poor function phenotypes: Reduced function of drug and increased statin associated myopathy risk	Reduced starting dose, adjust doses, combination therapy, consider drug-drug interactions, renal and hepatic function, and ancestry prior to statin treatment Cooper-DeHoff et al. (2022)
*IFNL3*	C/C, C/T, T/T (Reference C and variant T allele with rs12979860)	Favorable response: 79.1, Unfavorable response: 21				Pegylated interferon-α (PEG-IFN-α) and ribavirin (RBV)	Treat hepatitis C virus infection	Longer treatment time to eliminate the viral load and increased risk of side effects	Carefully decide the duration of treatment based on risk and benefits Muir et al. (2014)

Abbreviations: ND, not detected in the study cohort; NM, Normal Metabolizers; IM, Intermediate Metabolizers; PM, Poor Metabolizers; RM, Rapid Metabolizers; UM, Ultrarapid Metabolizers; TCAs, tricyclic antidepressants; SSRIs - selective serotonin reuptake inhibitors.

^a^ CYP4F2*4 allele was common in the Tiwi cohort with a high frequency of 0.4, carrying three genotypes *3/*4, *1/*4 and *4/*4 with 34%, 18% and 15% of prevalence respectively. However, according to PharmGKB the function of CYP4F2*4 is yet unknown.

^b^ (KNMP Pharmacist organization Guidelines, 2022).

Also, the CYP2C9*3 No function allele had a frequency of 0.42 in this cohort, but a lower frequency has been reported in other populations (Europeans: 0.08, South Asians: 0.1, Africans: 0.01, Oceanians: 0.02). The AF of CYP2C19*2 in Tiwi was 0.25, which was higher as compared to Europeans (0.15) and Africans (0.16) but lower than, that is, reported in Asians (0.3) and Oceanians (0.61). On the other hand, CYP2C19*3 had an AF of 0.12 in the Tiwi cohort but was much lower in other populations (<0.07) except in Oceanians (0.15). More than half of the Tiwi cohort carried alleles associated with impaired CYP2C19 function, with IMs contributing 46% and PMs 14.2%. The prevalence of IMs and PMs were similar in East Asians (IMs: 46% and PMs: 13%) but lower in other groups (IMs<41% and PMs<8%), except for PMs reported in Oceanians (57%) (Figure 3B, Supplementary Table S1). CYP2C19 is a predominant determinant of patient responses to clopidogrel, a widely used antiplatelet drug in individuals at a higher risk of myocardial infarction or stroke. The CYP2C19 IMs and PMs have been reported to exhibit reduced clopidogrel effectiveness, leading to treatment failures and a high risk of adverse cardiac and cerebrovascular events (Lee et al., 2022). The CYP2C19 PMs have shown greatly reduced tricyclic antidepressants (TCA) efficacy and decreased responses to selective serotonin reuptake inhibitors (SSRIs), proton pump inhibitors (PPIs), and the antifungal agent voriconazole, warranting dose adjustments or an alternative drug to avoid treatment failures and side effects (Hicks et al., 2015; Hicks et al., 2017; Moriyama et al., 2017; Lima et al., 2021) (Table 1).

Similarly, the prevalence of homozygous CYP2C9*3 in PMs was 17.9% in the Tiwi, which was comparatively higher than in other global populations (0.02%–1.2%). The CYP2C9*3 was found in heterozygous IMs (49.2%) and was highly prevalent in the Tiwi as compared to all other populations (3%–18% across global populations) (Figure 3B, Supplementary Table S1). The CYP2C9 IMs and PMs renders them vulnerable to toxicities related to nonsteroidal anti-inflammatory drugs (e.g., meloxicam, piroxicam), phenytoin, statins, siponimod, and warfarin therapy that might lead to serious bleeding. CPIC recommends dose adjustments and careful monitoring for ADRs or the use of an alternate drug not primarily metabolized by CYP2C9 in these groups (Johnson et al., 2017; Theken et al., 2020; Karnes et al., 2021; Cooper-DeHoff et al., 2022; KNMP Pharmacist organization Guidelines, 2022) (Table 1).

The CYP2D6*5 whole allele deletion was remarkably higher in Tiwi (0.24). A frequency of 0.17 and 0.16 have been previously reported among South Africans (Gaedigk and Coetsee, 2008) and north Indians (Taylor et al., 2020) respectively. The prevalence of *5 was comparatively lower among others, with 0.03 frequency in Europeans and 0.05 in Asians and Africans. The CYP2D6 enzyme is a key metabolizer for >25% of clinically used drugs (Zhou, 2009). We found that the CYP2D6*5 whole allele deletion was the major contributor in CYP2D6 PMs and IMs of the Tiwi cohort with 6.2% and 34.6% prevalence respectively. The presence of PMs and IMs were remarkably higher in the Tiwi as compared to other ethnicities except PMs reported in Europeans (PMs: 6.5% in Europeans and <3% in others; IMs: 20% in East Asians and <11% in others) (Figure 3B, Supplementary Table S1). Impaired CYP2D6 function has a high risk of ineffective analgesia and ADRs when conducting opioid therapy using codeine and tramadol in CYP2D6 IMs and PMs has been well established (Crews et al., 2021). In CYP2D6 IMs and particularly in PMs, reduced metabolism of the widely used classes of antidepressants including TCAs (Hicks et al., 2017) and SSRIs (e.g., fluvoxamine and paroxetine) (Hicks et al., 2015) has been frequently reported. CYP2D6 IMs and PMs have a higher risk of breast cancer recurrence when treated with tamoxifen (Goetz et al., 2018) and an impaired metabolism has been reported during atomoxetine treatment (Brown et al., 2019). Hence, genotype-guided treatment strategies in CYP2D6 phenotypes with an impaired function is recommended (Table 1). With 41% of the cohort being CYP2D6 IMs or PMs with a potentially impaired CYP2D6 metabolism, PGx studies of the Tiwi population thus have important implications for genotype-guided therapy related to several medications.

Another No function allele, NUDT15*3, was detected in Tiwi with an AF of 0.05, which was higher than Europeans (0.002) but lower than Asians (0.06). The NUDT15 IMs carrying the NUDT15*3 in *1/*3 genotype (9.4%) was lower than the prevalence reported in Asians (13%–17%) but much higher than previously reported in Europeans (0.76%) (Figure 3B, Supplementary Table S1). The NUDT15 plays an important role in the thiopurine response used in the treatment of inflammatory diseases and cancer. Reduced starting doses are recommended for IMs to prevent increased risk of thiopurine-related toxicities (Relling et al., 2019) (Table 1).

#### The prevalence of decreased function alleles and their clinical implications

Other functionally important alleles that predicted higher AF in the Tiwi population included the decreased function alleles in *CYP4F2* and *CYP2B6* associated with drug metabolism. The CYP2B6*6 had an AF of 0.39 in Tiwi, where lower frequencies were reported in other populations (<0.2) except in Africans (0.4) and Oceanians (0.6). Overall, 75.6% of the cohort predicted to have impaired CYP2B6 metabolism with the majority being IMs (50.5%). Other populations have reported a comparatively lower prevalence of 33%–47% for IMs. CYP2B6 PMs comprised 18%, which was higher than all others except in Oceanians (41%) and Africans (23.7%) (Figure 3B, Supplementary Table S1). Additionally, 7.2% of the Tiwi cohort showed rapid or ultrarapid metabolism of CYP2B6. CYP2B6 IMs and PMs face a greater risk of central nervous system toxicities upon administration of efavirenz, a commonly used drug in antiretroviral therapy, and CPIC recommends initiating treatments with lower doses to minimize complications (Desta et al., 2019) (Table 1).

Similarly, the CYP4F2*3 allele was found with an AF of 0.36 in this cohort. The prevalence of CYP4F2*3 in Tiwi was higher compared to Europeans (0.27), East Asians (0.23) and Africans (0.1) but closer to, that is, reported among Americans and South Asians (0.4). In contrast, decreased function alleles, *VKORC1* rs9923231 variant T allele and *ABCG2* rs2231142 variant T allele were reported with 0.1 and 0.03 frequency in the Tiwi cohort, associated with drug target and transport respectively. Global populations have reported higher frequencies of rs9923231(T) (AF ≥ 0.4) except for populations of South Asian and African ancestry (<0.2). Similarly, higher frequencies have been reported for rs2231142 (T) among East Asians (0.31) and Oceanians (0.2), while South Asians and Africans have reported lower frequencies of 0.09 and 0.006 respectively. Warfarin response is strongly associated with the rs9923231 variant in *VKORC1*, and lower doses are recommended for patients with affected genotypes (Johnson et al., 2017). This variant had a prevalence of 2% in homozygotes (rs9923231/rs9923231) and 14.2% in heterozygotes (*1/rs9923231) with a possible decreased function (Figure 3B, Supplementary Table S1). In addition to *CYP2C9* and *VKORC1* alleles, the CYP4F2*3 has also been linked to increased warfarin dose requirements in Europeans and Asians but not among Africans. The CYP4F2*3 impaired phenotype, predicted a prevalence of 11.33% in homozygotes and 14.16% in *1/*3 heterozygotes leading to possible decreased function phenotypes (Johnson et al., 2017) (Figure 3B, Supplementary Table S1). Also, the rs2231142 variant in *ABCG2*, which reduces the activity of the encoded transporter protein, was found to be heterozygous in 6.1% of the Tiwi cohort. Although not affected as rs2231142 homozygotes, dose adjustments based on disease and ancestry have been recommended for heterozygotes. The impaired ABCG2 has clinical implications with allopurinol which is used to reduce high blood uric acid levels requiring increased doses of the drug (Saito et al., 2016) (Table 1).

Additionally, we found two other decreased function alleles *6 and *80+*28 in *UGT1A1* gene, involved with drug metabolism which showed AF of 0.03 and 0.02 in the cohort respectively, with implications for atazanavir used in antiretroviral therapy (Gammal et al., 2016). The UGT1A1*6 allele has been reported with high frequency among East Asians (0.15) but much lower in Europeans (0.008).

#### Other functionally relevant alleles detected in Tiwi people

Some other functional pharmacogene variants found in Tiwi included the increased function allele SLCO1B1*20 associated with drug absorption which showed an AF of 0.11 (Europeans: 0.04). Also, the *IFNL3* variant T allele (rs12979860) involved with an unfavourable response had an 0.11 frequency in this cohort. A total of 20.9% of the Tiwi cohort were either homozygous or heterozygous for the rs12980275 variant allele in the *IFNL3* gene leading to an unfavourable response to pegylated interferon-α (PEG-IFN-α) and ribavirin (RBV) therapy in the treatment of hepatitis C infection, requiring careful consideration of the possibility of side effects before initiating therapy (Muir et al., 2014) (Table1). Also, several other variant alleles of interest were reported with high frequency among the Tiwi cohort. These included the CYP2D6*131 unknown function allele defined by the rs17002853 which was the second abundant *CYP2D6* allele detected in the cohort having an AF of 0.043. The CYP4F2*4 allele with an unknown function was also observed with 0.4 frequency in Tiwi which is defined by the two SNPs, rs3093105 and rs2108622.

For seven actionable pharmacogenes, *CFTR*, *RYR1*, *CACNA1S*, *DPYD*, *TPMT*, *G6PD*, and *CYP2C8,* over 99% of the individuals in the study cohort were predicted to have normal metabolism (Supplementary Table S2). For genes, *CFTR, TPMT, CACNA1S* and *RYR1* variant alleles were not detected within the Tiwi cohort. In *DPYD* gene, HapB3 (c.1236G>A) deficient allele was detected with 0.0012 frequency, which was 0.02 among Europeans and South Asians. Similarly, the CYP2C8*3 which had an AF of 0.001 was the only *CYP2C8* variant allele detected in Tiwi. The prevalence of CYP2C8*3 was 10%–23% among Caucasians while rare or absent in Asians and Africans (Ingelman-Sundberg et al., 2007). The *G6PD* deficient allele frequency within the cohort was low (0.011) with a high prevalence reported in malaria endemic regions, e.g., 33% of prevalence across Africa has been reported previously (Howes et al., 2012).

Overall, we identified that every Tiwi individual in the cohort carries at least one actionable genotype and 77% of them have at least three actionable genotypes with important implications for drug-gene interventions in the Tiwi population (Figure 3C).

### Prevalence of high-evidence PGx variants

**FIGURE 4:**
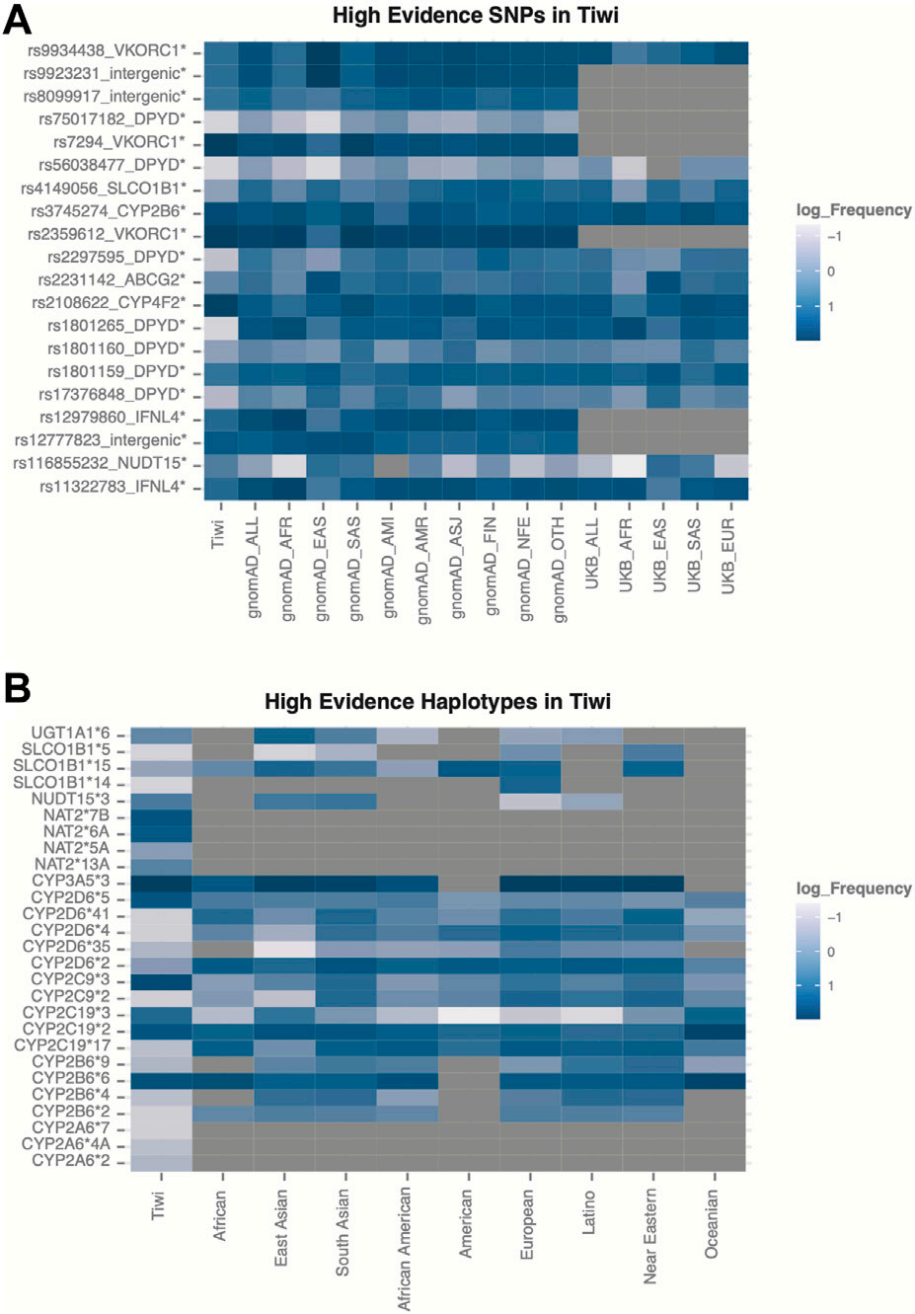
Variants with highest evidence for their pharmacological relevance identified in Tiwi. **(A)** Frequency distribution across global populations for the 20 high-evidence SNPs. Variants with statistically significant allele frequencies are marked with * (adj *p*-value <0.05 Vs. gnomAD_All) **(B)** Allele frequencies across global populations for the 27 high-evidence haplotypes in Tiwi.

Among the unique 265 variants in the PharmGKB with the highest level of evidence for their pharmacological relevance, we identified 20 high-evidence SNPs and 27 high-evidence variant haplotypes in the Tiwi cohort (Figure 4, Supplementary Table S3).

The frequency differences of the 20 high-evidence SNPs were significant (adj *p*-value <0.05 Vs. gnomAD_All) (Figure 4A). Of these, seven variants were common in Tiwi with an AF > 0.1 and two of them (rs7294, AF 0.9 and rs2108622, AF 0.78) had AFs two-fold higher compared to gnomAD_All population. Prevalences of these two variants were lower in all other populations (0.1–0.5), except rs7294, which was enriched in South Asians with an AF of 0.7. The intronic rs7294 T allele has been associated with increased warfarin doses compared to the reference C allele in different populations (Chen et al., 2014; Krishna Kumar et al., 2014). Overall, 347 and 65 Tiwi individuals carried the TT and CT genotype for this variant respectively, with potential implications for high warfarin dose requirements. Moreover, the association of the nonsynonymous variant rs2108622, the core variant in CYP4F2*3*,* with warfarin is well known (Johnson et al., 2017). The rs12777823 near *CYP2C18* was also common in Tiwi with an AF of 0.25 and the prevalence was higher in Africans and Asians (0.25–0.36) than other populations (0.1–0.2). The rs12777823 variant A allele has shown a significant interference with warfarin clearance and reduced doses are recommended in African Americans (Johnson et al., 2017). The rs2359612 in the *VKORC1* intronic region was prevalent in Tiwi with an AF > 0.9 compared to other populations. Also, the rs2359612 variant G allele has been reported with increased dose of warfarin in populations (Limdi et al., 2008; Schwarz et al., 2008). Non-synonymous core variant rs3745274 in *CYP2B6* showed an AF of 0.445 in Tiwi and usually reported high in Africans and South Asians (0.37–0.4), but lower in other global populations. As previously discussed, CYP2B6 is involved with efavirenz response. Intronic variant rs12979860 and splicing variant rs11322783 in *IFNL3/IFNL4* were common in Tiwi with an AF > 0.1, but lower compared to other populations. Both variants were associated with unfavourable response to PEG-IFN-α and RBV used in the treatment of hepatitis C infection (Muir et al., 2014).

Among the 27 high-evidence haplotypes identified in Tiwi, eight were common with prevalence >10%. As shown in Figure 4B, CYP2B6*6, CTP2D6*5, CYP2C19*2, CYP2C19*3, CYP2C9*3, CYP3A5*3, NAT2*6A, and NAT2*7B haplotypes showed the highest prevalence within the cohort, most of which have important drug interventions as detailed in Table 1.

### Spectrum of potentially actionable variants in VIPs

**FIGURE 5:**
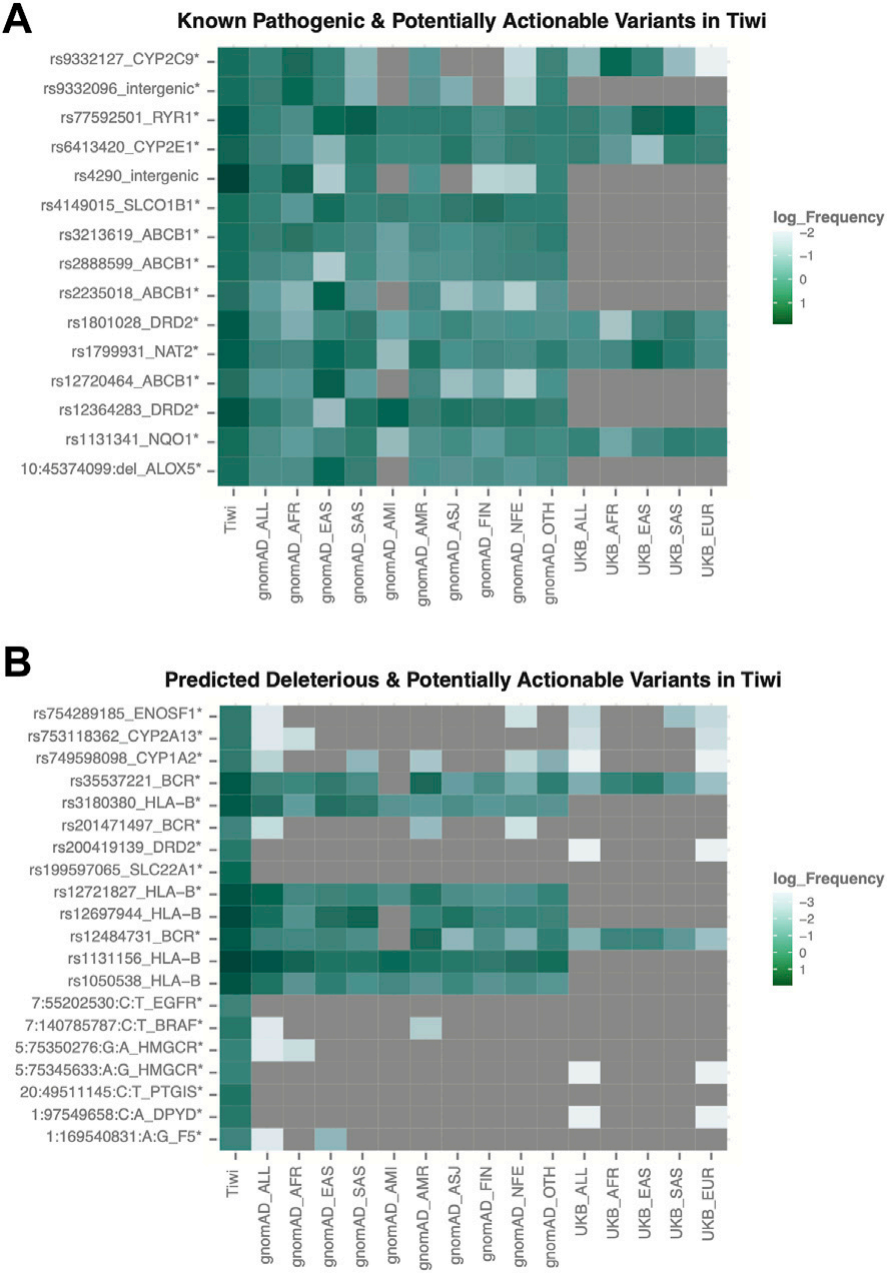
Frequency distribution of potentially actionable variants in VIP genes. **(A)** Rare pathogenic variants commonly enriched in Tiwi **(B)** Rare variants predicted to be deleterious and commonly enriched in Tiwi. Variants with statistically significant allele frequencies are marked with * (adj *p*-value <0.05 Vs. gnomAD_All). Variant rs12721827 was common in gnomAD_All (AF > 0.06).

We analysed 67 VIPs, defined by the PharmGKB to be significantly associated with drug metabolism with variants in these genes being associated with a higher risk of altered drug responses and serious side effects. In total, we identified 26,895 variants in the 67 VIPs, of which 300 were pathogenic or associated with drug response according to HGMD or ClinVar annotations. Among these, 14 rare variants were significantly enriched in Tiwi (Tiwi: >10%, gnomAD_All and UKB_All: <6%, adj *p*-value <0.05 Vs. gnomAD_All) (Figure 5A, Supplementary Table S3). The *CYP2C9* intronic variant, rs9332127 which is associated with warfarin sensitivity, was observed with 0.12 frequency in Tiwi, 0.16 average in Africans and less than 0.06 in all other populations. The prevalence of *SLCO1B1* upstream region variant, rs4149015 involved with increased pravastatin clearance was 0.13 in Tiwi and lower in all other populations except in East Asians and Finnish (East Asians: 0.13, Finnish: 0.11, others: <0.07). The rs4149015 variant was also inferred by star allele calling tools indicating the presence of a novel *SLCO1B1* allele in this population. Additionally, two nonsynonymous variants (rs1799931 and rs1131341) and intergenic rs9332096 variant were associated with reduced activity, while two upstream variants (rs6413420 and rs12364283) were associated with increased activity.

For 25,367 variants with no functional annotations in HGMD or ClinVar databases, their potential functional relevance was predicted using *in silico* functionality prediction algorithms. Interestingly, among the predicted 86 potentially deleterious variants, 31 exonic variants (30 nonsynonymous and one stop-gain) in 21 VIP genes were not reported in any major populations (gnomAD, 1KGP, UKB) or in public databases (dbSNP, HGMD, ClinVar) (Table 2, Supplementary Table S3). Of these 31 variants, five were common in Tiwi with AF > 1%. We observed another 16 nonsynonymous potentially deleterious rare variants (AF < 6% in gnomAD_All), which have been previously reported in either dbSNP or in one/more major populations, which were significantly enriched in Tiwi with AF > 20-fold compared to gnomAD_All or UKB_All populations (adj *p*-value <0.05 Vs. gnomAD_All) (Figure 5B, Supplementary Table S3).

**TABLE 2:** Novel potentially actionable variants detected in the Tiwi cohort.

Variant	Gene	Exonic function	Tiwi AF
^a^ **chr4:99287656:A:C**	***ADH1A***	**nonsynonymous SNV**	**0.07482**
^a^ **chr4:88139847:C:T**	***ABCG2***	**nonsynonymous SNV**	**0.038**
chr4:99318105:C:G	*ADH1B*	nonsynonymous SNV	0.00119
chr9:72918767:A:G	*ALDH1A1*	nonsynonymous SNV	0.001188
chr7:117642522:C:G	*CFTR*	nonsynonymous SNV	0.002375
^a^ **chr19:41010072:G:A**	***CYP2B6***	**nonsynonymous SNV**	**0.01069**
chr10:94942252:T:A	*CYP2C9*	nonsynonymous SNV	0.002375
chr10:94981215:G:A	*CYP2C9*	nonsynonymous SNV	0.002375
chr22:42128178:T:C	*CYP2D6*	nonsynonymous SNV	0.002632
chr7:99778079:C:G	*CYP3A4*	nonsynonymous SNV	0.002375
chr7:99780083:T:C	*CYP3A4*	nonsynonymous SNV	0.001188
chr7:99648354:G:T	*CYP3A5*	nonsynonymous SNV	0.00119
chr7:99672657:C:T	*CYP3A5*	nonsynonymous SNV	0.001188
chr1:169560659:T:C	*F5*	nonsynonymous SNV	0.002375
chr11:67584757:C:G	*GSTP1*	nonsynonymous SNV	0.001188
chr5:75351472:C:G	*HMGCR*	nonsynonymous SNV	0.001188
^a^ **chr8:18400234:G:A**	***NAT2***	**stop gain**	**0.01663**
chr16:69718136:A:G	*NQO1*	nonsynonymous SNV	0.004751
chr1:186680287:G:A	*PTGS2*	nonsynonymous SNV	0.003563
chr1:186676129:G:C	*PTGS2*	nonsynonymous SNV	0.002375
chr7:87628889:A:T	*RUNDC3B*	nonsynonymous SNV	0.002387
^a^ **chr19:38490101:G:C**	***RYR1***	**nonsynonymous SNV**	**0.08314**
chr19:38452829:A:G	*RYR1*	nonsynonymous SNV	0.007229
chr19:38473466:G:T	*RYR1*	nonsynonymous SNV	0.004762
chr19:38448702:G:T	*RYR1*	nonsynonymous SNV	0.002375
chr19:38494580:T:C	*RYR1*	nonsynonymous SNV	0.001188
chr19:38535149:G:A	*RYR1*	nonsynonymous SNV	0.001188
chr3:38633109:G:T	*SCN5A*	nonsynonymous SNV	0.002375
chr3:38633042:G:A	*SCN5A*	nonsynonymous SNV	0.001188
chr6:160122140:G:A	*SLC22A1*	nonsynonymous SNV	0.001188
chr18:662149:T:C	*TYMS*	nonsynonymous SNV	0.001188

a Bold text: Commonly detected in the Tiwi cohort with AF > 0.01 (1%).

## Discussion

The complexity of the human genome is strikingly highlighted by the existence of ethnically distinct pharmacogenomic variants that guides personalized medicine, with such diversity being particularly pronounced in geographically isolated groups (Rosenberg et al., 2005). Such population clusters are likely to possess a unique PGx profile distinct from major global populations. Non-Europeans carry the highest burden of functionally deleterious variants, many of which are not represented in current star allele definitions (McInnes et al., 2021). Moreover, rare genetic variants have been attributed greater functional significance, and identifying population-specific rare variants with pharmacological relevance is crucial to understanding the broader PGx profile of a population.

Most genetic and clinical PGx studies are based on populations of European ancestry, and the findings of these studies cannot be extrapolated to unique minority groups. Indigenous Australians are culturally and ancestrally diverse, comprising one of the largest Indigenous communities in the world. Despite many recent efforts to characterize their underlying genetic background, there have been insufficient pharmacogenetic studies focused on these Indigenous communities. Our study focuses on characterizing the PGx profile of the founder Indigenous population. We and others have characterized the blood group profiles (Jadhao et al., 2022), genetic susceptibility to chronic kidney disease (Thomson et al., 2019) and a variant affecting platelet function (Ningtyas et al., 2020) in this population. This study is the first to comprehensively investigate clinically actionable pharmacogenes in the Tiwi people, contributing novel, important findings and further validating the results of a previous attempt to characterise Tiwi PGx profiles (Jaya Shankar et al., 2022).

Our analysis of 459 Tiwi community members revealed that every individual in the study cohort carries at least one clinically actionable genotype associated with one or more of the 138 drugs linked to clinically actionable haplotypes across 19 pharmacogenes. Similar frequencies have been reported in other study groups across different gene panels. According to the PG4KDS study, nearly 98.5% Caucasian and 99.1% African people in the United States carry at least one high-risk genotype across 12 pharmacogenes (Dunnenberger et al., 2015). The eMERGE-PGx study across 82 pharmacogenes reported that 96.2% of the participants carry at least one actionable genotype respectively (Bush et al., 2016). These findings show the importance of selecting an appropriate gene panel for a specific population for pre-emptive testing, to ensure transferring the benefits to a majority.

Of 265 high-evidence variants in PharmGKB, 20 high-evidence SNPs and 27 high-evidence haplotypes were identified in the Tiwi cohort (Figure 4, Supplementary Table S3). Our findings also expand the current knowledge base, as we report an additional four pharmacologically important high-evidence variants, rs7294, rs2359612, rs11322783, and rs12777823 commonly enriched in Tiwi that were not observed among the known star alleles identified for this cohort. Furthermore, we identified another 14 functionally relevant known pathogenic variants (e.g., rs9332127), 16 potentially deleterious rare variants (e.g., rs3180380) (Figure 5, Supplementary Table S3), and 31 population-specific novel variants (Table 2) in VIP genes that could be potentially actionable in this population.

The results of our study have important implications for recommending suitable drugs and adjusting doses for this population based on the pharmacokinetics or pharmacodynamics of commonly prescribed drugs. We observed that nearly ten widely used drugs such as, clopidogrel, warfarin, atorvastatin, metoprolol, omeprazole, phenytoin, escitalopram, risperidone have clinically relavent drug-gene interactions reported in this study (Table 1). For example, we found that CYP2D6*5 whole allele deletion rates were nearly four-fold higher among the Tiwi people (24%) as compared to all other global populations. The CYP2D6*5 variant has been found at comparatively lower frequencies in other ethnic populations (Supplementary Table S1). Overall, 41% of the Tiwi people are either CYP2D6 IMs or PMs, with this rate being remarkably higher than all other reported global frequencies. Other CYP variants were also present at a relatively high frequency in the Tiwi cohort, much as in other populations. For example, the *CYP2C9* no-function alleles were detected at a higher prevalence in the Tiwi (42.5%) than in other global populations, with reported prevalence rates of 11% in South Asians, 7.6% in Europeans, and 1% in Africans. Furthermore, *CYP2B6* variants were present in 76% of the Tiwi cohort as compared to 20%–40% of African minority populations (Supplementary Table S1).

Variants in *CYP2C19*, *VKORC1*, and *CYP4F2* were significantly associated with warfarin response. Additionally, high-evidence variants such as rs7294, rs2359612, and rs12777823 and potentially actionable variants such as rs9332127 that were commonly identified in the Tiwi have important implications for warfarin responses, warranting the use of warfarin dosing algorithms to calculate the appropriate dose before treatment. Statin-induced myopathy risk was also predicted to be increased in more than half of the cohort due to variants in *CYP2C9*, *SLCO1B1*, and *ABCG2*. Moreover, we identified a commonly enriched potentially actionable variant, rs4149015, that is, related to pravastatin clearance. Also, responses to antidepressants such as TCAs and SSRIs were likely to be affected due to the presence of *CYP2D6* and *CYP2C19* variants in more than 50% of Tiwi people. In cases where a drug is affected due to variants in multiple genes, CPIC highlights the benefits of adjusting doses based on population-specific combined genotype information to improve the efficacy and safety of treatments. Affected Tiwi individuals also carry a higher risk of drug toxicities and markedly diminished efficacy in treatments related to a wide range of drugs or drug classes including analgesics (NSAIDs), antiplatelets (clopidogrel), antiepileptics (phenytoin), antiretrovirals (efavirenz), antifungals (voriconazole), PPIs (omeprazole, lansoprazole, dexlansoprazole), tacrolimus, and statins (atorvastatin, simvastatin, rosuvastatin, and fluvastatin), most of which are commonly used for the treatment of chronic kidney disease and associated complications prevalent among the Tiwi people. Overall, our results indicate that Tiwi people carry a unique PGx profile, providing a sound basis underscoring the importance of implementing a panel-based cost-effective PGx testing approach in this population prior to prescribing drugs whose metabolism might be affected. Ultimately, these findings are expected to enhance the quality of health for the Tiwi people while reducing the burden on healthcare systems due to ADRs, treatment failures and extended hospital stays. Our approach is adaptable and equally beneficial to people in other populations and healthcare systems globally.

There are a few limitations of this study. First, this study population is highly homogeneous. This is expected due to the isolation of the Tiwi people for more than 10,000 years. Although we eliminated three highly similar individuals (plink -king-cutoff 0.354) from downstream analysis, we are reluctant to remove a greater proportion due to the limited availability of genomic data and the social and ethical barriers to its collection. Therefore, some degree of relatedness bias is still represented in the population allele frequencies in our study. In addition, the availability of meta-information pertaining to diseases and the use of medications per individual is limited and we were thus not able to assess the actual proportion of the population that could benefit from our analysis, though such an analysis could have added an additional level of evidence regarding the importance of PGx testing in this population. Moreover, we employed an ensemble genotyping approach to identify star alleles. Ensemble genotyping approaches have previously reported high haplotype concordance (Twesigomwe et al., 2020) and have shown better capability to detect star alleles using WGS data with sufficient coverage ( ≥ 30x) (Lee et al., 2022). Due to the highly polymorphic nature of certain genes, presence of complex structural variants, and paralogs within close proximity have significantly challenged accurate calling of star alleles in genes such as *CYP2D6* and *CYP2B6*. Despite these factors, our findings show that pre-emptive testing can provide valuable guidance for clinical decision-making aimed at avoiding unfavourable responses following treatment and enhancing therapeutic efficacy.

## Data Availability

The Tiwi genomic data presented in this article cannot be readily accessed due to ethical approval restrictions on sharing the data publicly. Requests to access the datasets should be directed to SN (shiv.nagaraj@qut.edu.au).
